# Growth performance, biochemical and haematological parameters of BALB/c mice fed on staple grains and bee larvae (*Apis Mellifera*) blended complementary foods

**DOI:** 10.1016/j.heliyon.2022.e09003

**Published:** 2022-02-24

**Authors:** Shewangzaw Addisu Mekuria, John N. Kinyuru, Beatrice Kiage Mokua, Mesfin Wogayehu Tenagashaw

**Affiliations:** a Department of Food Science and Nutrition, Jomo Kenyatta University of Agriculture and Technology, P.O. Box 62000-00200, Nairobi, Kenya; b University of Gondar, P.O. Box 196, Gondar, Ethiopia; c Applied Human Nutrition Chair, Bahir Dar University, P.O. Box 26, Bahir Dar, Ethiopia

**Keywords:** Biochemical, Complementary foods, Growth, Haematological, Mice, Treatments

## Abstract

In Sub-Saharan Africa, inadequate complementary feeding practices and being nutritionally inadequate are primary factors in infant and young child malnutrition, growth failure, and high morbidity and mortality. Therefore, novel complementary foods need to be developed to alleviate malnutrition problems in IYC. Therefore, this experimental study aimed to assess the effects of newly developed grain-bee larvae blended complementary foods on the growth performance, haematological, and biochemical parameters of BALB/c mice. A complete randomized design was used and a total of 75 BALB/c mice were assigned to each of the five treatments. The treatments were: T1 = Casein diet; T2 = 57 % Maize, 29 % Teff, 14 % Soybean; T3 = 58 % Maize, 29 % Teff, 13 % Bee larvae; T4 = Commercial wean mix; and T5 = Basal diet alone. The *in vivo* experiment trial was done for 28 days along with seven days of adaptation. Dietary intake was not significantly different (P = 0.96) between treatments, but it was noted that T3 had gained the highest final body weight (38.52 g). The examined biochemical parameters showed T4 had the lowest serum protein (6.27 mg/dl) and globulin (3.61 mg/dl). Compared to others, T3 significantly (P < 0.001) increased WBC (4 × 10^6^ mm^3^), RBC (11.37 × 10^3^ mm^3^), Haemoglobin (16.42 g/dl), and Hematocrit (63.04 %). The highest serum levels of zinc (0.55 mg/dl) and iron (2.08 mg/dl) were reported on T2, while the highest serum calcium content (10.64 mg/dl) was reported on T1. The results indicated that T3 can aid body growth, health, and prevent malnutrition in infants and young children.

## 1 Introduction

Malnutrition is a global problem in infants and children, that affects the world population. According to the reports of, UNICEF (2021), children under five affected by stunting and wasting are 21.3 and 2.1%, respectively. People living in those developing nations, including in Sub-Saharan African (SSA) or South Asian countries, are frequently considered seriously malnourished (Raza et al., 2020; Tao and Li, 2018). Therefore, undernutrition remains a significant problem in developing countries. Malnourished children have a greater risk of infection, ill-health, and mortality, and early growth retardation is associated with distinct variants of negative functional outcomes, including reduced cognitive function, delayed motor development, and poor school performance. According to recent studies, undernutrition accounts for around half of all mortality in children below the age of five (UNICEF, 2021).

In many developing countries, including SSA, poor complementary infant and young children (IYC) feeding practices and nutritional deficiency are key contributors to malnutrition, growth failure, and high morbidity and mortality (Mutisya et al., 2021). Malnutrition of varying degrees is associated with feeding infants unhealthy and low-quality Complementary Foods (CFs) (Abiose et al., 2015; Jeelani et al., 2020). Malnutrition due to poor complementary feeding practices is a serious concern in many low-income countries when CFs are composed of starch-based grains (Oladiran and Emmambux, 2020) that provide insufficient protein and micronutrients but adequate energy (Dewey, 2003), which reflect on hinders an individual's health (Tian et al., 2016). Protein - Energy Malnutrition (PEM) is a prevalent childhood disorder (Ikpeme-Emmanuel et al., 2009; Jee, 2021) and is primarily caused by a deficiency of energy, protein, and micronutrients (Batool et al., 2015). It occurs during the critical transitional period of weaning in infants, stunting their physical and mental development (Ahmed et al., 2020; Ryckman et al., 2021). Thus, the situation can be prevented to a large extent by introducing CFs of quality and quantity at the right time in the right proportions (Reddy et al., 1990). Protein-energy malnutrition results in widespread changes in both physiological functions and haematological systems (Khan et al., 2020; Warrier et al., 1990; Waterlow, 1972). The most common haematological change in IYC with PEM is anemia (Anticona and San Sebastian, 2014), with iron deficiency being the major cause of anemia (Özkale et al., 2014). Micronutrient deficiencies (Beal et al., 2021), specially zinc deficiency, cause stunting of growth, anemia, and greater susceptibility to infection (Bhutta et al., 2008; Ramesh et al., 2016). Feeding sufficient, nutritious, and safe CFs can thus to prevented IYC from the risk of malnutrition (FAO, 2017).

Starch-based foods are the major staple food in most developing countries (Ryckman et al., 2021), as animal protein food is very expensive (Cao and Li, 2013; Kinyuru. et al., 2015) resulting in an inadequate protein intake for normal body growth and development (Obatolu et al., 2000; Schönfeldt and Gibson Hall, 2012). Protein consumption is expected to rise in the future as the world's population grows (Haber et al., 2019; Henchion et al., 2017). Traditional CFs that are mainly cereal-based are nutritionally deficient (Suri et al., 2014). Cereals and legumes are important sources of calories and protein due to their availability, low cost, and acceptability. According to the report findings by Tenagashaw et al. (2017), soybean, teff, and maize are among those readily available local food crops that can complement each other and meet the recommended daily food allowance for growing infants. Bee larvae (*Apis Mellifera*) are nutrient-dense foods with high nutritional content (Ghosh et al., 2016; Jensen et al., 2019), and are developed as a component of CFs (Shewangzaw et al., 2021). Several studies have shown honey bee brood contains 35 % protein Ghosh et al. (2016) and 45.70 g/100g Shewangzaw et al. (2021) on a dry matter basis. Therefore, the protein content of *A. Mellifera* is comparable to that of other protein sources, including chicken, beef, and pork, which have a protein content of 54.7, 40.5, and 27.7%, respectively.

To alleviate malnutrition problems associated with IYC, novel CFs from insects, such as the one from a combination of bee larvae and soybean with stable grains, need to be developed. However, little is known about the potential physiological effects of newly developed CFs on humans. Mice feeding experiments are needed to determine biomarkers suitable for human intervention research and are further liable to apply to study physiological aspects, such as nutrient absorption, metabolism, and function of the newly developed CFs. Feeding experiments comparing organic and commonly developed food are conducted to assess the overall health impact of the animals as a model for the results experienced by human consumers (Velimirov et al., 2010). The concentration of blood components shows nutrients in the food for blood cells synthesis and the nutritional status of IYC. Therefore, this experimental study assessed growth performance, organ weight, haematological, biochemical, and serum mineral bioavailability of BALB/c mice fed on the innovative stable grains – *A. mellifera* based CFs and commercial wean-mix food.

## 2 Materials and methods

### 2.1 Diet formulation and preparation

All aspects of animal care and experimentation in this study followed the National Institutes of Health Guide for the Care and Use of Laboratory Animals and followed the EEC directive of 1986 (86/609/EEC) and were approved by the ethical committee of the College of Veterinary Medicine and Animal Sciences, University of Gondar, Ethiopia. Five iso-nitrogenous (10 %) experimental mouse diets were developed as follows. The basal diet was formulated, according to the methods by Adeoti and colleagues (Adeoti et al., 2018), and used as a control. In brief, the basal diet contained a mixture of corn starch (610 g/kg), wheat bran (50 g/kg), vegetable oil (100 g/kg), mineral and vitamin premix (50 g/kg), glucose (60 g/kg), oyster shell (20 g/kg), sucrose (88 g/kg), bone meal (20 g/kg), and NaCl (2 g/kg). The casein diet and commercial wean-mix were purchased from the local supermarket in Ethiopia. The food ingredients bee larvae (*A. mellifera*) was collected from the modern beehives in the Gondar university apiary farm, Ethiopia, and other grains soybean seed (*Glycine max*), red teff (*Eragrostis tef (Zucc.)*), and maize (*Zea mays L.)* were purchased from the local market and Agricultural Research Center, Gondar, Ethiopia. Then, the two experimental complementary diets were developed using NutriSurvey software (version, 2007), i.e., diet 1 composed of 57 % maize, 29 % teff, and 14 % soybean, whereas diet 2 was composed of 58 % maize, 29 % teff, and 13 % bee larvae and extrusion process. After the flour was blended, the following extrusion parameters were considered: moisture content (17 %), barrel temperature (150 °C), and screw speed with a 29 g/min feed rate, using a pilot-scale twin-screw extruder (model Clextral, BC-21 No. 124, Clextral, Firminy, France) (Shewangzaw et al., 2021). The nutritional composition of the commercial and developed complementary diets was analyzed and is stated in Table 1.Where Diet 1- maize (57 %), teff (29 %), and soybean (14 %); Diet 2- maize (58%), teff (29 %), and bee larvae (13 %).

**Table 1:** Proximate analysis of experimental diets and commercial wean mix (g/100 g), energy content (kcal/100 g), and mineral composition (mg/100 g) (on a dry matter base).

Nutrients	Diet 1	Diet 2	Commercial wean-mix
Moisture	4.41 ± 0.19	5.72 ± 0.17	2.46 ± 0.39
Ash	2.09 ± 0.09	1.88 ± 0.04	2.01 ± 0.08
Protein	12.56 ± 0.17	11.75 ± 0.15	10.78 ± 0.29
Fat	12.4 ± 0.1	14.3 ± 0.1	2.82 ± 0.36
Fiber	4.52 ± 0.04	3.47 ± 0.08	2.75 ± 0.17
Carbohydrate	64.02 ± 0.41	62.87 ± 0.23	79.19 ± 0.55
Energy	417.93 ± 3.23	427.18 ± 2.42	385.25 ± 1.77
Fe	40.17 ± 0.38	40.94 ± 0.29	5.79 ± 0.16
Zn	2.84 ± 0.18	2.92 ± 0.16	2.32 ± 0.11
Ca	31.78 ± 0.11	44.34 ± 0.49	68.20 ± 0.12

The mice experimental diets were formulated to obtain a 10 % protein level and blended with the basal diet, which is presented in Table 2 (Adejuwon et al., 2021; Adeoti et al., 2018; Adepoju and Ajayi, 2016; Osundahunsi and Aworh, 2003). The composition of the experimental food treatments was calculated using Eq. (1) Adeoti et al. (2018).

(1) $$IN=\frac{a}{100}\times b=\frac{10}{100}\times c$$

Where:

**Table 2:** Experimental treatments food formulation for BLAB/c mice model.

Treatments	Protein Content (%)	Wt. of Basal Diet (g/kg)	Wt. of Food (g/kg)	Final Protein level (%)
Treatment 1	99	898.99	101.01	10
Treatment 2	12.56	203.82	796.18	10
Treatment 3	11.75	148.94	851.06	10
Treatment 4	10.78	72.36	927.64	10
Treatment 5	10	1000	_	10

a = The original sample protein content of as analyzed

b = Required weight of sample for the new food blend

c = The total weight of the blend

IN = Isonitrogenous

The treatments are: Treatment 1- casein diet; Treatment 2- soybean (14 %), teff (29 %), and maize (57 %); Treatment 3- bee larvae (13 %), teff (29 %), and maize (58 %); Treatment 4- commercial wean mix; Treatment 5- basal diet alone. Finally, a group of mice was randomly assigned to one of the experimental treatments.Where Treatment 1- Casein diet + Basal Diet; Treatment 2- maize (57 %), teff (29 %), and soybean (14 %)+ Basal Diet; Treatment 3- maize (58%), teff (29 %), and bee larvae (13 %) + Basal Diet; Treatment 4- Commercial wean mix + Basal Diet; Treatment 5- Basal diet alone; wt = weight.

### 2.2 Animals and housing

A total of 75 pathogen-free male, BALB/c mice, an average 26–28 days old with 31.57 g of average body weight, were obtained in the School of Pharmacy, University of Gondar, Ethiopia. For the experimental trials, the animal sample size was calculated using the standard formula (Arifin and Zahiruddin, 2017). The mice were reared in cages made of polypropylene, measuring 22.2 cm × 30.80 cm × 16.24 cm, with a wireframe-grade stainless steel feeder and fitting for a leak-proof water bottle. The experimental animal was randomly assigned to five groups of fifteen mice each. The experimental treatment was conducted in triplicate with five mice per cage in a complete randomized design. Each mouse and cage was given an identification mark. The *in vivo* experiment was done for 28 days, with an additional seven days of adaptation under the same conditions of environmental temperature (26 ± 0.42 °C) and relative humidity (55 ± 5%), with a 12:12 h light-dark cycle. Clean tap water and food were offered *ad libitum* during the experiment. Cage's bedding sawdust material was changed once a week, while water was changed daily for 28 days.

### 2.3 Feed intake and growth performance

The feeding and watering activities of all mice were observed every day. Data on the amount of feed offered and leftovers from each group of mice were recorded. The weekly body weight of each animal was measured and recorded before the start of feeding. Growth performance was monitored and calculated in terms of feed intake, weight gain, and feed conversion ratio, which was calculated as in Eq. 2, Eq. 3, and Eq. (4), respectively (Marwa et al., 2019) where:

(2) Feed Intake (FI) = (Feed offered − Feed leftover)

(3) Weight Gain (WG) = (Final body weight – Initial body weight)

(4) Feed Conversion Ratio (FCR) = Feed Intake/Weight Gain

The percentage of body weight gain Eq. (5) was calculated as follows (AL-Shinnawy, 2009):

(5) $$\text{Body ​Weight ​Gain ​}\left(\text{\%}\right)\text{ ​}=\text{ ​}\frac{\text{Mean ​final ​body ​weight ​}-\text{ ​Mean ​initial ​body ​weight}}{\text{Mean ​initial ​body ​weight}}\text{ ​× ​}100$$

### 2.4 Blood sample collection and analysis

After a 12-hr fast, all mice were sacrificed on the twenty-eighth day of the experimental period. Before termination, the mice were anaesthetized (ketamine-xylazine anaesthesia (mixed in the ratio of 4:1)) with 0.25 ml/100 g body weight intraperitoneally and then terminated by cardiac puncture injection (Parasuraman et al., 2017; Santos et al., 2016). Blood was collected, and immediately administered into the tube with anticoagulant ethylene di-amine tetra acetic acid (EDTA) bottles and serum containers, then hand-mixed several times and kept on a wet icebox for further haematological analyses (Pierre et al., 2011). Plasma was immediately separated using a laboratory centrifuge (TD4C dc brushless motor centrifuge, Hunan, China) centrifugation (3000 rpm at 4 °C for 10 min) and frozen in aliquots at −20 °C, while organs (liver, heart, kidney, and spleen) were immediately excised and weighed.

#### 2.4.1 Haematological and biochemical analysis

Aliquot of blood sample was immediately subjected to a full blood count, which includes important haematological parameters, namely, White Blood Cells (WBC), Red Blood Cells (RBC), Haemoglobin (HGB), Hematocrit (HCT), Platelet Count (PLT), Mean Cell Haemoglobin (MCH), Mean Cell Volume (MCV), and Mean Cell Haemoglobin Concentration (MCHC), using a fully automated ABX MICROS Pentra 60C + Analyzer (Horiba ABX, Montpellier, France) Mazzaccara et al. (2008). The biochemical test was conducted briefly, and 50-μl blood samples were aspired and distributed to the various chambers for sample analysis. Total protein, and serum albumin, evaluation, was done using an automated pentra (C400, France) blood analyzer.

#### 2.4.2 Serum analysis for zinc, iron, and calcium

A total of 1-ml of serum was mixed with 1 ml of concentrated nitric acid and 0.5 ml hydrogen peroxide in propylene tubes. The mixture was maintained at 60 °C for 2 h to allow digestion of the samples until a clear, colorless solution was achieved. The digest thus obtained was diluted by adding 2.5 ml of deionized water. The sample solutions were then centrifuged at 2000 rpm for 5 min and subsequently analysed (Luna et al., 2019). Different concentrations (0.5, 1.0, 2.0, 5.0, and 10.0 mg/L) of stock solutions of trace elements were used for calibrating standard graphs. Finally, the concentrations of zinc (Zn), iron (Fe), and calcium (Ca) were read using AAS (a Buck 210 VGP, U.S.A) with their hollow cathode lamp at their respective wavelengths of 213.9 nm, 248.3 nm, and 422.7 nm, respectively (AOAC, 2006).

### 2.5 Statistical analysis

Data on growth performance, haematological, biochemical, serum mineral bioavailability were expressed as means and standard deviations. The SPSS software windows (version, 23.0) were used for this analysis. The obtained data were subjected to one-way analysis of variance (ANOVA), and Tukey's HSD test was used for multiple means comparisons. A p-value of less than 0.05 was considered statistically significant.

## 3 Results and discussion

### 3.1 Feed intake and body weight of experimental mice

The first one thousand days of an infant's life are the most critical time in child development. Hence, complementary feeding practices should be improved to maximize children's potential for growth and development (Martorell, 2017). Inappropriate complementary feeding results in malnutrition due to a mismatch in nutritional requirements of intake (Keller, 2019). Our study showed that (Table 3) at the end of the experiment, the final body weight (g), weight gain (g), body weight gain (%), and Feed Conversion Ratio (FCR) were significant differences among the dietary groups. There was no statistically significant difference between treatments in feed intake (P > 0.05) or initial body weight (P > 0.05), but a significant difference (P < 0.01) in final body weight. The mice's nutritional status showed that the mice fed with T2 (38.39 g) and T3 (38.52 g) had better growth performance than those fed with T1 (37.15 g), T4 (35.02 g), and T5 (33.37 g). Similarly, the weight gain (g/day) of the mice was significantly different (P < 0.01) and ranged from 1.67 to 7.05, the highest being on T2 (7.05 g) and T3 (6.63 g). The increase in final body weight of T2 and T3 might be attributed to the type of nutrients in the diets that were easily absorbed in the body (Obatolu et al., 2000).

**Table 3:** Average feed intake and body weight of experimental BALB/c mice.

Parameters	Experimental Groups					P-Value
	T1	T2	T3	T4	T5	
Feed Intake (g)	64.41 ± 11.72	66.70 ± 17.66	67.91 ± 12.86	58.71 ± 18.57	60.53 ± 21.64	>0.05
Initial body weight (g)	31.61 ± 1.08	31.35 ± 1.08	31.89 ± 0.42	31.31 ± 0.13	31.70 ± 1.22	>0.05
Final Body Weight (g)	37.15 ± 1.06^a^	38.39 ± 0.66^a^	38.52 ± 0.66^a^	35.02 ± 2.35^b^	33.37 ± 2.48^b^	0.01
Weight Gain (g)	5.53 ± 2.23^ab^	7.05 ± 1.63^a^	6.63 ± 1.06^a^	3.69 ± 1.40^b^	1.67 ± 1.64^b^	0.01
Body weight gain (%)	17.53 ± 4.23^bc^	22.61 ± 6.05^a^	20.83 ± 3.63^a^	11.79 ± 4.47^c^	5.28 ± 5.08^c^	0.03
FCR	12.34 ± 2.65^c^	9.30 ± 1.25^e^	10.24 ± 1.57^d^	15.91 ± 6.32^b^	36.25 ± 13.97^a^	0.02

The values are expressed as mean ± SD. Means with different alphabets in the same row are significantly different (P < 0.05); FCR = Feed Conversion Ratio; AWG = Average Weight Gain; T1 = Casein diet, T2 = maize, teff, and soybean, T3 = maize, teff, and bee larvae, T4 = commercial wean mix, T5 = Basal diet.

Results of body weight gain (%) revealed that there was a significant difference (P < 0.05) between experimental treatments. A high body weight gain percentage was recorded on T2 (22.61) and T3 (20.83), and a low recorded on T4 (11.79) and T5 (5.28). The high percentage of body weight gain on T2 and T3 can be due to the high protein ingredients included in the diet i.e., soybean (12.56 g/100g) and bee larvae (11.75 g/100g) Shewangzaw et al. (2021). According to Adeoti et al. (2018), the quality and quantity of consumed protein could influence weight gain. A similar trend was observed in FCR. The FCR is a measure of how well an animal converts feed intake (feed usage) into live body weight (Adeyemi et al., 2015). The study confirmed a significant difference (P > 0.01) in FCR between treatments. Mice fed with T2 (9.3) exhibited a higher FCR than T3, T1, T4, and T5, which were 10.24, 12.34, 15.91, and 36.25, respectively. From the observed results of the mouse model, both T2 and T3 CFs would support the growth of IYC. Therefore, both T2 and T3 may be suitable as CFs, specially to support infant growth and development and prevent malnutrition among under-five-aged children.

**Figure 1:**
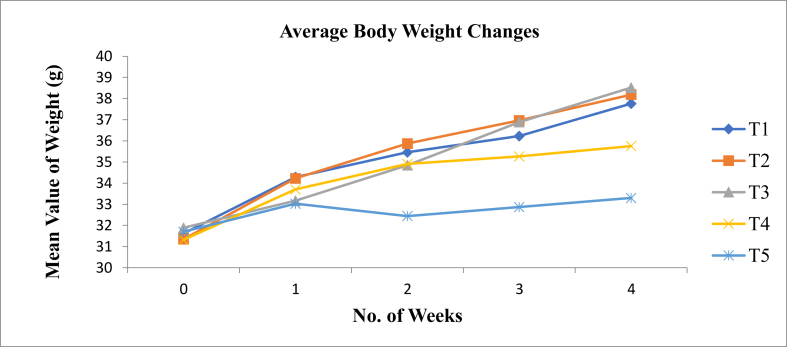
Trends of average body weight change of experimental BALB/c mice over twenty-eight days. Were T1 = Casein diet, T2 = Maize, teff, and soybean, T3 = Maize, teff, and bee larvae, T4 = Commercial wean mix, T5 = Basal diet. From the initial experimental week to the end of the experimental period, there was an increment in body weight in all experimental treatments. T1, T2, and T3 had similar trends of body weight change over time; however, T4 and T5 increased steadily, specially T5, whose body weight change was very slight.

Trends in the average body weight change of experimental animals over twenty-eight days were observed (Figure 1). The growth trends of mice fed both T1 and T4 were significantly lower than those fed T2 and T3 but had a greater increase than T5 (basal diets). The difference in growth trend between treatments would be because of the quality of proteins, i.e., the inclusion of soybean and *A. mellifera* in the diets and their intake. According to the findings (Adejuwon et al., 2021; NRC, 1995), protein deficiency in young animals results in reduced growth, anemia, hypoproteinemia, depletion of body protein, muscular wasting, emaciation, and, if sufficiently severe, death.

### 3.2 Organ weights

Bodyweight changes commonly accompany internal organ weight variations, making understanding organ weight differences more complex (Bailey et al., 2004). There were no statistically significant differences (P > 0.05) in organ weights (kidneys, liver, and heart) between treatments (Table 4). The results in this study agreed with the findings of Osundahunsi and Aworh (2003). There were no significant differences in the weights of the liver and kidney of rats that received the complementary diets compared to those mice fed on basal and casein diets. The lower weight of organs in rats fed a basal diet indicated an abnormal development. However, the spleen weight (g) was a significant difference (P < 0.001) between the groups that received the experimental treatments, which ranged between 0.29 and 0.53 g. High weight of the spleen was observed on T2 (0.53 g), followed by T1, T3, T4, and T5, which were 0.35, 0.29, 0.31, and 0.29 g, respectively. The difference in spleen weight between treatments would be aligned with the positive correlation of body weight. Reports of (Bailey et al., 2004; Eisen, 1986; Simpson and Spears, 1973) showed a positive genetic correlation between body weight and respective organ weights. Also, there was an association of RBC size with enlargement of the spleen (Pivkin et al., 2016). However, the weight of spleen change can be difficult to interpret (Resendez and Rehagen, 2017).

**Table 4:** Results of organ weight (g) of experimental BALB/c mice.

Organs	T1	T2	T3	T4	T5	P-Value
Kidneys	0.68 ± 0.11	0.70 ± 0.11	0.70 ± 0.11	0.61 ± 0.06	0.62 ± 0.08	0.16
Liver	2.69 ± 0.34	2.69 ± 0.46	2.42 ± 0.45	2.43 ± 0.17	2.37 ± 0.11	0.13
Heart	0.23 ± 0.04	0.23 ± 0.05	0.22 ± 0.04	0.22 ± 0.44	0.18 ± 0.04	0.06
Spleen	0.35 ± 0.05^b^	0.53 ± 0.05^a^	0.29 ± 0.15^b^	0.31 ± 0.13^b^	0.29 ± 0.08^a^	<0.001

The values are expressed as mean ± SD. Means with different alphabets in the same row are significantly different (P < 0.05); T1 = Casein diet, T2 = maize, teff with soybean, T3 = maize, teff with bee larvae, T4 = commercial wean mix, T5 = Basal diet.

### 3.3 Biochemical and haematological parameters

The examination of blood allows investigating several metabolites and other constituents in the bodies of animals and plays a vital role in the physiological and nutritional status of an organism (Etim et al., 2014). The biochemical assessment uses laboratory measurements of serum protein and serum micronutrient levels, which are parameters for assessing general nutritional status and identifying specific nutritional deficiencies (Bartlett, 2004; Gilbert and Weiner, 2013). Adding to the previous parameters, a haematological analysis was conducted in this study to check the effects of varied diets on blood parameters. Table 5 shows the effects of experimental diets on the biochemical and haematological properties of BALB/c mice fed extruded complementary food samples compared with casein, commercial wean mix, and basal diets.

**Table 5:** Effects of nutritional intervention on biochemical and haematological parameters of BALB/c mice.

Parameters	T 1	T 2	T 3	T 4	T 5	P-Value
**Biochemical**						
Serum Protein (mg/dl)	8.37 ± 0.12^bc^	8.80 ± 0.36^b^	7.08 ± 0.72^cd^	6.27 ± 0.84^d^	10.78 ± 0.39^a^	<0.001
Serum Albumin (mg/dl)	2.94 ± 0.09	3.01 ± 0.12	2.94 ± 0.14	2.65 ± 0.21	3.12 ± 0.22	>0.05
Serum Globulin (mg/dl)	5.43 ± 0.09^bc^	5.79 ± 0.42^b^	4.14 ± 0.80^cd^	3.61 ± 0.79^d^	7.66 ± 0.48^a^	<0.001
**Haematological**						
WBC (×10^6^ mm^3^)	5.27 ± 0.95^ab^	2.77 ± 0.67^bc^	4.0 ± 0.30^b^	1.97 ± 0.38^c^	3.57 ± 0.21^b^	<0.001
RBC (×10^3^ mmv^3^)	9.72 ± 0.12^b^	7.32 ± 0.41^c^	11.37 ± 0.57^a^	2.73 ± 0.44^d^	9.09 ± 0.22^b^	<0.001
HGB (g/dl)	13.27 ± 0.46^b^	11.34 ± 0.60^c^	16.42 ± 0.66^a^	4.97 ± 0.80^d^	12.4 ± 0.60^bc^	<0.001
HCT (%)	48.80 ± 0.10^bc^	41.55 ± 0.66^d^	63.04 ± 0.55^a^	13.40 ± 0.44^e^	47.10 ± 0.26^c^	<0.001
PLT (×10^3^ mm^3^)	740.10 ± 23.27^a^	100.13 ± 1.02^c^	165.37 ± 1.52^c^	392.27 ± 4.31^b^	187.07 ± 1.41^c^	<0.001
MCV (μm^3^)	53.00 ± 1.00	53.67 ± 2.08	54.67 ± 0.58	54.67 ± 1.53	52.67 ± 1.98	>0.05
MCH (pg)	14.67 ± 0.25^b^	14.50 ± 0.20^b^	14.37 ± 0.40^b^	16.50 ± 0.30^a^	13.53 ± 0.35^c^	<0.001
MCHC (g/dl)	25.87 ± 2.25^b^	27.67 ± 1.53^b^	26.50 ± 2.17^b^	32.20 ± 0.89^a^	25.60 ± 0.98^b^	<0.004
Red Cell Distribute (RDW) (%)	16.17 ± 0.87^d^	17.07 ± 0.25^cd^	17.53 ± 0.67^abcd^	17.97 ± 0.42^abc^	18.97 ± 0.76^a^	<0.004
Mean Platelet Volume (MPV) (μm^3^)	7.73 ± 0.47^b^	6.30 ± 0.26^c^	6.53 ± 0.35^c^	6.63 ± 0.38^c^	9.53 ± 0.31^a^	<0.001
Platelet Distribution Width (PDW) (%)	5.87 ± 1.53^b^	1.97 ± 1.90^bc^	2.33 ± 2.14^bc^	12.93 ± 0.70^a^	1.43 ± 1.24^c^	<0.001

The values are expressed as mean ± SD. Means with different alphabets in the same row are significantly different (P < 0.05); T1 = Casein diet, T2 = maize, teff, and soybean, T3 = maize, teff, and bee larvae, T4 = commercial wean mix, T5 = Basal diet; WBC = White Blood Cells; RBC = Red Blood Cells; HGB = Haemoglobin; HCT = Hematocrit; PLT = Platelet Count; MCV = Mean Cell Volume; MCH = Mean Cell Haemoglobin; and MCHC = Mean Cell Haemoglobin Concentration.

The biochemical profiles of the mean serum albumin (mg/dl) were not significantly different (P = 0.10) between the experimental treatments. Albumin is the most abundant protein in human serum (Keller, 2019). However, serum protein and globulin were significantly different (P < 0.001) between experimental treatments. Serum protein and globulin (mg/dl) revealed that there were high amounts of serum protein (10.78), globulin (7.66) on T5, and small amounts on T4, which was 6.27 serum protein, and 3.61 globulin. A high total serum protein level showed dehydration, and a low level showed that protein is not being digested or absorbed properly and hence could lead to malnutrition (Wu, 2016). Therefore, based on the *in vivo* experiment, when the mice were inadequately fed, there was a risk of stunted growth and a range of biochemical changes that could impair development to a large extent (Amankwah et al., 2010).

Also, blood haematological parameters were an indicator of nutritional status and assessed the disease of IYC. Haematological profiles of WBC, RBC, HGB, HCT, PLT, MCH, MPV, and PDW were significantly different (P < 0.001) between experimental treatments. The WBC (×10^6^ mm^3^) results were significantly different (P < 0.001) between experimental treatments. WBC plays an important role in the formation of disease barriers and, therefore, be involved in the formation of antibodies to protect the body against pathogens (Amara et al., 2020). Thus, from this finding, mice intake of T4 had low amounts of WBC (1.97), while T1 had high amounts (5.27). Therefore, the intake of T1 has increased the number of leukocytes and has increased disease resistance. Soetan et al. (2013), reported animals with low WBC were exposed to a high risk of disease infection, while those with high levels could generate antibodies in the means of phagocytosis and have high resistance to diseases.

A similar trend in RBC, HGB, and HCT was observed between experimental treatments. Results of RBC (×10^3^ mm^3^) and haemoglobin (gm/dl) were an indicator of anemia. The RBC was significantly different (P < 0.001) between experimental treatments. The study showed that T4 had low RBC (2.73 × 10^3^ mm^3^) amounts than that of T1, T2, T3, and T5, which were 9.72, 7.32, 11.37, and 9.09, respectively. Mice that were fed with T4 would be susceptible to anemia because of the low level of RBC. According to the findings AL-Shinnawy, 2009; da Silva Lopes et al. (2018) decreased RBC was seen in anemia of any cause. Likewise, HGB is another confirmatory parameter for anemia. Testing was the primary method of anemia diagnosis (Kariyeva et al., 2003). Results of HGB (g/dl) concentration showed that T5 (16.42) had high amounts and was low in T4 (4.97). The results of HCT were significantly different (P < 0.001) between experimental animals. The high HCT percentage was recorded on T3 (63.04 %) and low on T4 (13.40 %). The increased HCT percentage was shown either due to an increase in the number of RBCs or a slowing in circulating plasma volume (Chineke et al., 2006), with better transportation of oxygen and absorption of nutrients (Etim et al., 2014). According to the report of (Peters et al., 2011) HCT, HGB, and mean corpuscular HGB are major parameters for evaluating circulatory erythrocytes and are significant in the diagnosis of anaemia.

The MCV was not significantly different (P > 0.05) between experimental treatment groups. From the observed experiment, T4 had the highest records of MCH (16.56 pg) and MCHC (32.20 g/dl), which indicated anaemia (Åsberg et al., 2014). Blood platelets are implicated in blood clotting. The findings of PLT (×10^3^ mm^3^) showed that a significant difference (P < 0.001) between experimental treatments was observed and ranged from 100.13 to 740.10. The highest PLT (×10^3^ mm^3^) counts were observed on T1 (740.10) and followed T4 (392.27), T5 (187.07), T3 (165.37), and T2 (100.13). The low PLT concentration showed that the process of clot formation (blood clotting) would be prolonged, resulting in excessive loss of blood with injury (Etim et al., 2014). The biochemical and haematological profiles of experimental mice varied because of several factors, such as gender, age, genetic variation, diet, and environmental conditions (Santos et al., 2016). Therefore, in this study, the variation might be due to diet differences.

### 3.4 Serum mineral bioavailability

Table 6 shows the effect of the experimental diets on serum Zn, Fe, and Ca content. Serum, Zn, and Ca were significantly different (P < 0.001) between experimental treatments. Similarly, serum Fe was significantly different (P < 0.05) between experimental treatments. High concentrations of serum Zn were observed on T2 (0.55 mg/dl) and T3 (0.62 mg/dl). The reason might be associated with the protein quality of the foods. According to the findings of (Willoughby and Bowen, 2014), Zn intake was closely related to dietary protein intake. The bioavailability of serum Fe has been observed ranging from 0.83 to 2.08 mg/dl in the experimental animals. The high amount of serum Fe on T2 (2.08 mg/dl) was due to the inclusion of soybean legume (Çakir et al., 2019) and red teff (Baye, 2014), which might have contributed iron to the CFs. The importance of adequate Fe and Zn intakes for older infants was recognized to avoid the development of iron deficiency growth faltering and anemia (Krebs, 2001). Minor elements like Fe and Zn have long been linked to hunger, mental capacity growth, functioning, and growth in young infants (Lee and Yang, 2017). The deficiencies of certain micronutrients, such as iron, result in potentially irreversible negative effects on brain development along with other damaging psychological outcomes (Lozoff et al., 2006). However, mice intake of T4 was observed in low amounts of serum Zn (0.09 mg/dl) and Ca (0.26 mg/dl). The low Zn deficiency was mainly due to the inadequate intake or low absorption from the diet (Tian et al., 2016). According to the findings of Keller (2019), a deficiency of zinc would cause anorexia, skin lesions, impaired visual function, diarrhea, anemia, decreased lymphocyte function, and mental retardation. In addition, stunted children exhibited lower blood zinc levels, and lower food intakes were linked with anorexia caused by zinc inadequacy in stunted children compared to non-stunted children (Gibson et al., 2007).

**Table 6:** Bioavailability of serum minerals (Zn, Fe, and Ca) of experimental BALB/C mice (mg/dl).

Minerals	T1	T2	T3	T4	T5	P-Value
Zn	0.26 ± 0.04^b^	0.55 ± 0.02^a^	0.62 ± 0.03^a^	0.09 ± 0.04^c^	0.22 ± 0.04^b^	<0.001
Fe	1.25 ± 0.42^abc^	2.08 ± 0.42^a^	1.17 ± 0.13^abc^	1.67 ± 0.42^abc^	0.83 ± 0.42^c^	0.02
Ca	10.64 ± 1.03^a^	5.51 ± 0.13^b^	2.18 ± 0.26^c^	0.26 ± 0.13^d^	0.31 ± 0.04^d^	<0.001

The values are expressed as mean ± SD. means with different alphabets in the same row are significantly different (P < 0.05); Zn = Zinc; Fe=Iron; Ca=Calcium; T1 = Casein diet, T2 = maize, teff with soybean, T3 = maize, teff with bee larvae, T4 = commercial wean mix, T5 = Basal diet.

On T5, low serum Fe (0.83 mg/dl) and Ca (0.31 mg/dl) were recorded. Low intakes of Fe were consistent with a high prevalence of anemia seen between the ages of 6 and 24 months (Lutter and Rivera, 2003). The highest serum Ca (10.64 mg/dl) recorded on T1 can be due to the high content of calcium in the casein diet. According to the report of (Bennett et al., 2000), the efficiency of Ca absorption was enhanced due to increased dietary casein intake. Usually, micronutrient deficiencies can occur during the weaning period, because infants have higher nutrient demands relative to their increased energy requirements (Qasem et al., 2015). Therefore, CFs that contain optimal levels of specific nutrients, such as iron and zinc (Campoy et al., 2018) should be provided for IYC.

## 4 Conclusion

This study shows that intake of the newly developed CFs, particularly the bee larvae-based CFs, increased the bodyweight of young mice and therefore could be explored for use as infant CFs as a cost-effective way to prevent malnutrition. Based on the biochemical and haematological results, intake of the commercial wean mix was likely to expose mice to disease infection and anaemia. Compared to the soybean-based and commercial CFs, the bee larvae-based CFs improved body weight and both haematological parameters such as WBC, RBC, HGB, HCT, MCH, MCHC, and the bioavailability of Zn. Therefore, the bee larvae-based CFs could be suitable CFs specifically to support infant growth and development and prevent malnutrition in IYC using the mouse model. In addition, however, a clinical study of the intake of the newly developed CFs on the physiological and biological effects of infants and young children is required.

## Declarations

### Author contribution statement

Shewangzaw Addisu Mekuria: Conceived and designed the experiments; Performed the experiments; Analyzed and interpreted the data; Contributed reagents, materials, analysis tools or data; Wrote the paper.

John N. Kinyuru: Conceived and designed the experiments; Performed the experiments; Analyzed and interpreted the data; Wrote the paper.

Beatrice Kiage Mokua, Mesfin Wogayehu Tenagashaw: Performed the experiments; Analyzed and interpreted the data; Wrote the paper.

### Funding statement

This work was supported by the DAAD/RUFORUM, In-Country/In-Region Scholarship Programme–Regional Universities Forum for Capacity Building in Agriculture (2018), Germany (57429563).

### Data availability statement

Data included in article/supplementary material/referenced in article.

### Declaration of interests statement

The authors declare no conflict of interest.

### Additional information

No additional information is available for this paper.
